# Metabolic Profiling Reveals Biochemical Pathways and Potential Biomarkers of Spinocerebellar Ataxia 3

**DOI:** 10.3389/fnmol.2019.00159

**Published:** 2019-06-27

**Authors:** Zhi-hua Yang, Chang-he Shi, Li-na Zhou, Yu-sheng Li, Jing Yang, Yu-tao Liu, Cheng-yuan Mao, Hai-yang Luo, Guo-wang Xu, Yu-ming Xu

**Affiliations:** ^1^Department of Neurology, The First Affiliated Hospital of Zhengzhou University, Zhengzhou University, Zhengzhou, China; ^2^CAS Key Laboratory of Separation Science for Analytical Chemistry, Dalian Institute of Chemical Physics, Chinese Academy of Sciences, Dalian, China

**Keywords:** spinocerebellar ataxia 3, SCA3, metabolomics, pathways, biomarkers

## Abstract

Spinocerebellar ataxia 3, also known as Machado-Joseph disease (SCA3/MJD), is a rare autosomal-dominant neurodegenerative disease caused by an abnormal expansion of CAG repeats in the *ATXN3* gene. In the present study, we performed a global metabolomic analysis to identify pathogenic biochemical pathways and novel biomarkers implicated in SCA3 patients. Metabolic profiling of serum samples from 13 preclinical SCA3 patients, 13 symptomatic SCA3 patients, and 15 healthy controls were mapped using ultra-high-performance liquid chromatography-mass spectrometry and gas chromatography-mass spectrometry techniques. The symptomatic SCA3 patients showed a metabolic profile significantly distinct from those of the preclinical SCA3 patients and healthy controls. The principal differential metabolites were involved in the amino acid (AA) metabolism and fatty acid metabolism pathways. In addition, four candidate serum biomarkers, FFA 16:1 (palmitoleic acid), FFA 18:3 (linolenic acid), L-Proline and L-Tryptophan, were selected to discriminate between symptomatic SCA3 patients and healthy controls by receiver operator curve analysis with an area under the curve of 0.979. Our study demonstrates that symptomatic SCA3 patients present distinct metabolic profiles with perturbed AA metabolism and fatty acid metabolism, and FFA 16:1, FFA 18:3, L-Proline and L-Tryptophan are identified as potential disease biomarkers.

## Introduction

Spinocerebellar ataxia 3 (SCA3) or Machado-Joseph disease (MJD) is the most common of the SCAs, with a worldwide prevalence of 1.5 cases per 100,000 individuals (Ruano et al., 2014). It is caused by an abnormal expansion of CAG repeats in the *ATXN3* gene and is characterized by a wide range of clinical features, including progressive ataxia, spasticity, ophthalmoplegia, and extrapyramidal signs (Durr et al., 1996; Riess et al., 2008; Schmitz-Hübsch et al., 2008; Jacobi et al., 2011; Costa Mdo and Paulson, 2012; Paulson, 2012). The median age of disease onset is about 40 years, and the patients usually die within 15–20 years (van de Warrenburg et al., 2005; Rub et al., 2013). Unfortunately, the pathogenesis mechanisms of SCA3 are not fully elucidated and no current therapeutic approach can alleviate the symptoms effectively (Evers et al., 2014; Li et al., 2015; Wu et al., 2015).

An increasing body of evidence indicates that the preclinical stage of SCAs already presents with detectable non-ataxia signs, including oculomotor deficits in SCA3, slowing of saccade in SCA7 and SCA2, and impaired smooth pursuit eye movements (SPEMs) in SCA17 (Globas et al., 2008; Maas et al., 2015; Wu et al., 2017). Thus, the preclinical stage may provide a window for disease intervention. Although some disease-modifying compounds have emerged in clinical trials, sensitive biomarkers to measure subtle therapeutic benefits are still lacking (Schulte et al., 2001; Saute et al., 2014, 2015). Therefore, identification of molecular pathways and biomarkers may prove beneficial in uncovering pathogenic mechanisms, identifying drug targets, monitoring disease progression, and assessing therapeutic effects (Lima and Raposo, 2018).

Metabolomics has emerged as a powerful technique, which explores the metabolic responses towards internal or external stimuli by comprehensively monitoring the variations in small molecules in certain biological samples (Nicholson and Lindon, 2008). Alterations in brain function can directly impact the biofluid composition in which metabolites are in dynamic equilibrium. Metabolites in the biofluid reflect the chemical imbalances in the cerebrospinal fluid (CSF) at the brain level, or in the blood and urine at the systemic level (Lima and Raposo, 2018). Metabolomics has been widely developed to map potential perturbations and identify novel biomarkers in neurodegenerative disease, such as Alzheimer’s disease, Parkinson’s disease, motor neuron disease, Huntington’s disease (Zhang et al., 2013; Chen-Plotkin, 2014; Jové et al., 2014). Iorio et al. (1993) investigated the serum fatty acid profile using gas chromatographic analysis in patients with Friedreich’s ataxia and SCA and found no significant differences in the fatty acid profiles of these patients. Griffin et al. (2004) defined a metabolomic phenotype in the brain of a SCA3 mouse model using ^1^H-NMR and identified an increase in the glutamine concentration and a decrease in the myo-inositol concentration in the brain. More recently, Toonen et al. (2018) performed a metabolomic analysis of the plasma in a SCA3 mouse model and identified tryptophan as the most promising biomarker. However, metabolomic analysis has not yet been reported in SCA3 patients.

Herein, we sought to determine the biochemical pathways and potential biomarkers in SCA3. A global metabolomics approach using the metabolomics platforms of ultra-high-performance liquid chromatography-mass spectrometry (UHPLC-MS) and gas chromatography-mass spectrometry (GC-MS) was used to analyze the serum samples of preclinical and symptomatic SCA3 patients.

## Materials and Methods

### Subjects

A cohort of 26 genetically confirmed SCA3 patients were enrolled from the First Affiliated Hospital of Zhengzhou University, including 13 preclinical SCA3 patients and 13 symptomatic SCA3 patients. All patients underwent a detailed neurological examination by two neurological specialist doctors. The severity of ataxia was evaluated using the Scale for the Assessment and Rating of Ataxia (SARA) and International Cooperative Ataxia Rating Scale (ICARS). Symptomatic SCA3 was defined as proven SCA3 mutation with symptomatic ataxia (SARA ≥ 3). Pre-SCA3 was defined as proven SCA3 mutation with mild coordination deficits (SARA < 3), and/or unspecific neurological symptoms (Schmitz-Hübsch et al., 2006; Maas et al., 2015; Lima and Raposo, 2018). The Mini-Mental State Examination (MMSE) was used to measure the general cognitive function. Non-ataxia features were also evaluated, including muscle cramps, sensory disturbances, hyperreflexia or hyporeflexia in lower limbs, extrapyramidal signs, extensor plantar, impaired vibration sense. Meanwhile, 15 age-, sex-, and BMI-matched volunteers without any neurological or psychiatric diseases were enrolled as healthy controls. The CAG repeats of *ATXN3* were tested using capillary electrophoresis (Souza et al., 2016).

All the serum samples were collected and stored using standard procedures (Yin et al., 2013; Kamlage et al., 2014). All the participants in this study did not take any medications or any irritation causing drink/food 72 h before the test. Peripheral venous blood was collected in 5 ml K^+^ -EDTA anticoagulant tubes (Sarstedt) in the morning after an overnight fasting. The blood was gently mixed and allowed to clot for 30 min in a 37°C water bath. Serum was extracted by centrifuging at 5,000 rpm for 10 min (4°C), and was stored at −80°C immediately until analysis.

The Ethics Committee of the First Affiliated Hospital of Zhengzhou University approved this study. Written informed consent was obtained from each participant.

### UHPLC-MS Analysis

An aliquot of 200 μl methanol containing internal standards was fully mixed with 50 μl serum, in order to remove the protein. The suspension was subsequently drawn and lyophilized. The lyophilized powder was resuspended in 50 μl 20% acetonitrile in water by a 30 s vortex. After centrifugation, the supernatant was directly used for UHPLC (Waters, Milford, MA, USA) coupled to Q Exactive HF MS (Thermo Fisher Scientific, Waltham, MA, USA) analysis. For electrospray ionization positive (ESI+) mode, BEH C8 column (100 mm × 2.1 mm, 1.7 μm; Waters, Milford, MA, USA) was used for separation. The Mobile phases were 0.1% formic acid in water (A) and 0.1% formic acid in acetonitrile (B). The gradient started with 10% B and was maintained for 1 min, subsequently increased to 40% B within 5 min, and then reached 100% at 17 min. After maintaining for 5 min, it returned to the initial 10% B. For electrospray ionization negative (ESI−) mode, HSS T3 (100 mm × 2.1 mm, 1.8 μm; Waters, Milford, MA, USA) column was employed with 6.5 mM NH_4_HCO_3_ in water (C) and 6.5 mM NH_4_HCO_3_ in 95% methanol (D) as the mobile phases. The gradient started with 0% D and was maintained for 1 min, increased linearly to 40% D at 2 min and subsequently to 100% D at 13 min. After being maintained for 6 min, the gradient returned to the initial 0% D. For both the modes, the column temperature was set at 50°C with elution flow rate 0.35 ml min^−1^. For both ion modes, the MS capillary temperature was 300°C with the auxiliary air heating temperature 350°C. The sheath gas and auxiliary gas flow rate were set as 45 and 10 units. Full scan resolution was set as 12 million. For the positive mode, m/z scan range was 80–1,200 Dalton and the spray voltage was 3.5 kV. For the negative mode, m/z scan range was 70–1,200 Dalton and the spray voltage was 3 kV.

### GC-MS Analysis

An aliquot of 400 μl methanol containing internal standard was fully mixed with 100 μl serum to remove the protein and extract metabolites. After centrifugation at 15,000 *g* and 4°C for 15 min, 360 μl of the supernatant was lyophilized. An aliquot of 100 μl methoxyamine pyridine solution (20 mg ml^−1^) was added into the lyophilized powder. After 30 s vortex and 15 min ultrasonication, oximation was performed at 40°C in a water bath for 2 h. Subsequently, 80 μl of N-methyl-N-(trimethylsilyl) trifluoroacetamide (MSTFA) was added for the following silylation, which lasted for 1 h at 40°C in a water bath. After centrifugation, the supernatant was ready for subsequent GC-MS analysis. Prepared sample (1 μl) was injected into the GCMS-QP 2010 analysis system (Shimadzu, Kyoto, Japan) with the split ratio of 10:1. DB-5 MS capillary column (30 m × 250 μm × 0.25 μm; J & W Scientific, Folsom, CA, USA) was used for metabolites separation. The detailed separation parameters have been reported in our previous work (Ye et al., 2014). The full scan mode (33–600 m/z) was chosen with the event time modified as 0.5 s.

To ensure the data quality, quality control (QC) samples were prepared by mixing all the samples. During the analysis of the samples, one QC sample was run after every 10 injections.

### Data Analysis

For both the UHPLC-MS and GC-MS raw data, peak alignments were first performed and the ion tables exported. Unique ions for known metabolites were chosen and the derived final metabolite tables were imported into GC-MS solution software (Shimadzu, Kyoto, Japan) and TraceFinder software (Version 3.2, Thermo Fisher Scientific, Rockford, IL, USA) for batch integration. The ion structure elucidation was performed with the National Institute of Standards and Technology (NIST) library for GC-MS database and an in-house LC-MS2 library for UHPLC-MS database.

The clinical parameters were expressed as means (SD, standard deviation). Statistical analysis was carried out using Chi-square test, one-way analysis of variance (ANOVA) test, and student’s *t*-test by SPSS software (Version 20.0, IBM Corporation, Armonk, NY, USA). Significant differences were indicated at levels of* p* < 0.05.

The annotated metabolites from UHPLC-MS (ESI+, ESI−) and GC-MS analysis platforms were combined for further analysis. Multivariate analysis was performed to visualize general clustering of the samples using SIMCA-P+ software (Version 13.0, Umetric, Umea, Sweden). We performed unsupervised analysis by Principal Component Analysis (PCA), and supervised analysis by Orthogonal Projections to Latent Structures Discriminant Analysis (OPLS-DA) to assess the classification of the samples. Cross-validation was performed to check the robustness of the constructed OPLS-DA model. Variable importance in Projection (VIP) in the OPLS-DA was used to identify metabolites that mainly contributed to the separation between two groups. Metabolites with VIP > 1.0 were chosen for the subsequent Wilcoxon−Mann−Whitney test. To correct for multiple testing, false discovery rates (FDR) were calculated using *q* values. Metabolites with both multivariate significance and univariate significance (VIP > 1.0 and *q* < 0.05) were considered as the differential markers. The potential association between the candidate metabolites and clinical characteristics in SCA3 patients was also evaluated using Pearson correlation test by SPSS software.

To further explore the related metabolic pathway disruptions, MetaboAnalyst 3.0^1^, Human Metabolome Database (HMDB^2^), and Kyoto Encyclopedia of Genes and Genomes (KEGG^3^) were employed.

The scatter plots of the candidate biomarkers were generated using GraphPad Prism version 7.0. Receiver Operating Characteristic curve (ROC) analysis and Logistic regression analysis were performed using SPSS software to evaluate the predictive potential of the candidate diagnostic biomarkers.

## Results

### Clinical Characteristics of the Participants

The clinical characteristics of preclinical SCA3 patients, symptomatic SCA3 patients, and healthy controls are summarized in Table 1. The three groups were comparable for sex, age, BMI, MMSE and CAG repeat numbers, while the scores of SARA and ICARS are significantly different. For the non-ataxia features, muscle cramps could be detected in 30.7% (4/13) of pre-SCA3 carriers and 38.4% (5/13) of the symptomatic SCA3 patients, sensory disturbance in 15.4% (2/13) of pre-SCA3 carriers and 53.8% (7/13) of the symptomatic SCA3 patients, hyperreflexia in the lower limbs in 23.1% (3/13) of pre-SCA3 carriers and 61.5% (8/13) of the symptomatic SCA3 patients, hyporeflexia in the lower limbs in 15.4% (2/13) of pre-SCA3 carriers and 30.7 (4/13) of the symptomatic SCA3 patients, extrapyramidal signs in 15.4% (2/13) of pre-SCA3 carriers and 53.8% (7/13) of the symptomatic SCA3 patients, extensor plantar reflex and impaired vibration sense in 53.8% (7/13) and 7.7% (1/13) of symptomatic SCA3 patients, respectively, while none in pre-SCA3 carriers.

**Table 1:** The clinical characteristics of the participants.

Characteristics	Pre-SCA3 (*n* = 13)	SCA3 (*n* = 13)	Con (*n* = 15)	*P*
Sex (Female)	7	6	6	0.76^a^
Age (years)	33.8 (7.2)	39.2 (7.5)	34.3 (7.3)	0.13^b^
BMI (kg/m^2^)	20.3 (2.4)	21.9 (3.4)	22.4 (3.5)	0.20^b^
Disease duration (years)	NA	8.2 (4.4)	NA	NA
CAG repeat length	69.3 (3.7)	71.5 (3.3)	NA	0.40^c^
MMSE	29.2 (0.3)	29.7 (0.5)	29.9 (0.4)	0.55^b^
SARA score	1.1 (1.0)	13.7 (3.6)	0.0 (0.0)	<0.001^b^
ICARS score	3.1 (3.0)	22.6 (6.6)	0.0 (0.0)	<0.001^b^
Non-ataxia features				
Muscle cramps (%)	30.7	38.4	0	0.032^a^
Sensory disturbance (%)	15.4	53.8	0	0.002^a^
Hyperreflexia in lower limbs (%)	23.1	61.5	0	<0.001^a^
Hyporeflexia in lower limbs (%)	15.4	30.7	0	0.04^a^
Extrapyramidal signs (%)	15.4	53.8	0	0.006^a^
Extensor plantar (%)	0	53.8	0	<0.001^a^
Impaired vibration sense (%)	0	7.7	0	0.005^a^

*Pre-SCA3, preclinical SCA3; SCA3, spinocerebellar ataxia type 3; Con, controls; SARA, scale for the assessment and rating of ataxia; ICARS, international cooperative ataxia rating scale; MMSE, mini-mental state examination; NA, not applicable. Values are presented as Mean (SD); ^a^Chi-square; ^b^*ANOVA*; ^c^t-test*.

### Metabolic Overviews of the SCA3 Patients

Three independent metabolomics platforms, including UHPLC-MS (ESI+), UHPLC-MS (ESI−), and GC-MS, were employed to collect comprehensive metabolic data. QC samples were prepared for monitoring the instrument robustness during data acquisition. The relative standard distribution (RSD) of the ions detected in each platform is shown in Supplementary Figure S1. Typical total ion chromatograms (TIC) from the QC are shown in Supplementary Figure S2. The results indicated good reproducibility and stability during the procedure.

**Figure 1:**
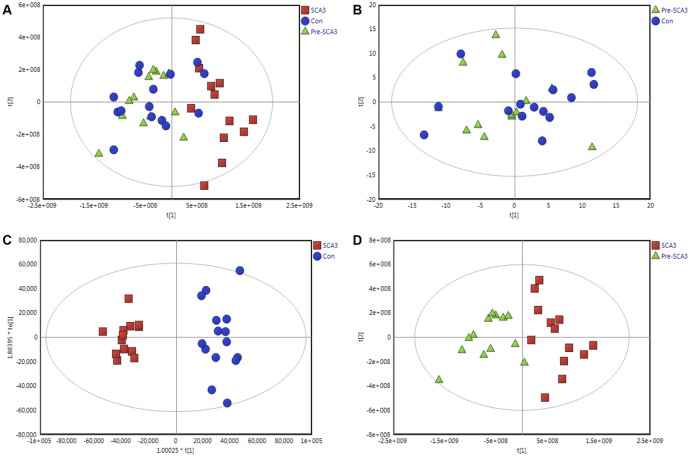
Multivariate analysis of Pre-spinocerebellar ataxia 3 (SCA3) group, symptomatic SCA3 group and control group. **(A)** Principal component analysis (PCA) scores plot of symptomatic SCA3 group, Pre-SCA3 group, and control group. **(B)** PCA scores plot of Pre-SCA3 group and control group. **(C)** OPLS-DA model of symptomatic SCA3 group and control group. **(D)** OPLS-DA model of symptomatic SCA3 group and Pre-SCA3 group. Green triangles, Pre-SCA3 group; Red squares, symptomatic group; Blue dots, control group.

The data were qualified for the following metabolomics data analysis. Based on the integrated metabolomics analytical data, 321 metabolites in total were identified, including 115 metabolites from UHPLC-MS (ESI+), 62 metabolites from UHPLC-MS (ESI−) and 144 metabolites from GC/MS (Supplementary Table S1). In the PCA plot of the three groups, the SCA3 group was separated significantly from the control and Pre-SCA3 groups, while Pre-SCA3 group and control group overlapped (Figure 1A, R^2^X_cum_ = 0.93, Q^2_cum^ = 0.86). The PCA plot of Pre-SCA3 group and control group showed no significant distinction (Figure 1B, R^2^X_cum_ = 0.249, Q^2_cum^ = 0.057).

To further identify the metabolites that discriminate the SCA3 group and Pre-SCA3 or control group, supervised OPLS-DA models were performed between two groups (Figures 1C,D). The variables were unit variance scaled and cross-validation with 200-time permutation tests were used to identify the reliability of the models. The R^2^Y_cum_ and Q^2^Y_cum_ of the OPLS-DA model for SCA3 group and control group were 0.945, 0.794 with four components responsible for the classification. The R^2^Y_cum_ and Q^2^Y_cum_ of the OPLS-DA model for SCA3 group and Pre-SCA3 group were 0.851 and 0.691 with two components.

### Differential Metabolites Related to SCA3

Univariate statistical analysis was subsequently performed based on the VIP of OPLS-DA model. Metabolites with VIP >1 in OPLS-DA and *q* value < 0.05 in the Wilcoxon-Mann-Whitney test after correction for multiple testing were selected as significantly differential metabolites. In total, 18 differential metabolites were highlighted between groups (Table 2). The correlation analysis showed negative correlations between the SFA levels and ICARS scores (*r* = −0.714; *p* = 0.006), FFA 16:0 levels and ICARS scores (*r* = −0.649; *p* = 0.016), and a positive correlation between L-proline levels and MMSE scores (*r* = 0.593; *p* = 0.033; Table 3).

**Table 2:** The differential metabolites between SCA3 group, and Pre-SCA3 group, control group.

Metabolites	Analysis	SCA3/Con			SCA3/Pre-SCA3		
	platforms	VIP^a^	*q*^b^	FC^c^	VIP^a^	*q*^b^	FC^c^
MUFA	ESI−	8.36	<0.0001	1.79	7.98	<0.0001	1.93
SFA	ESI−	5.06	0.0001	0.82	5.16	<0.0001	0.82
PUFA	ESI−	6.97	<0.0001	1.52	6.70	<0.0001	1.60
FFA 16:0	ESI−	1.85	0.046	0.93	-	-	-
FFA 16:1	ESI−	2.29	0.0016	1.93	2.40	<0.0001	2.24
FFA 18:0	ESI−	4.37	<0.0001	0.64	4.14	<0.0001	0.63
FFA 18:1	ESI−	7.95	0.0001	1.78	7.50	<0.0001	1.90
FFA 18:2	ESI−	6.86	0.0001	1.59	6.55	<0.0001	1.67
FFA 18:3	ESI−	1.23	0.0134	1.45	1.27	0.0076	1.47
GCDCA	ESI−	1.20	<0.0001	0.20	1.55	0.0001	0.23
L-Valine	ESI+	1.28	0.0144	0.86	-	-	-
L-Leucine	ESI+	1.42	0.0436	0.88	-	-	-
L-Tryptophan	ESI+	2.39	0.0017	0.78	1.80	0.0390	0.86
L-Tyrosine	ESI+	1.03	0.0015	0.72	-	-	-
L-Phenylalanine	ESI+	2.66	0.0014	0.83	-	-	-
L-Proline	ESI+	2.32	0.0249	0.40	1.07	0.0083	0.70
Acetylcarnitine	ESI+	2.24	0.003	1.49	2.29	<0.0001	1.61
Hippuric acid	ESI−	-	-	-	1.77	0.0001	0.37

*FFA, free fatty acid; GCDCA, glycochenodeoxycholate; MUFA, monounsaturated fatty acid; SFA, saturated fatty acid; PUFA, polyunsaturated fatty acid. ^a^Variable importance in the projection (VIP) was obtained from OPLS-DA. ^b^*q* values were calculated from Wilcoxon-Mann-Whitney test, and corrected for multiple testing from false discovery rates. ^c^FC: fold change, calculated from the arithmetic mean values of each group*.

**Table 3:** Pearson’s correlation analysis between metabolite levels and clinical characteristics in symptomatic SCA3 patients.

Metabolites		MUFA	SFA	PUFA	FFA 16:0	FFA 16:1	FFA 18:0	FFA 18:1	FFA 18:2	FFA 18:3	GCDCA	L-Valine	L-Leucine	L-Trypto phan	L-Tyro sine	L-Phenyl alanine	L-proline	Acetylcar nitine
Age	*r*	0.355	−0.48	0.085	−0.193	0.255	−0.462	0.356	0.127	−0.135	−0.5	−0.431	−0.281	−0.264	−0.066	−0.514	−0.076	0.06
	*p*	0.233	0.097	0.782	0.528	0.4	0.112	0.232	0.679	0.66	0.082	0.141	0.352	0.384	0.829	0.072	0.805	0.846
BMI	*r*	−0.055	−0.001	0.034	0.087	−0.275	−0.103	−0.012	0.02	−0.014	−0.006	−0.416	−0.085	−0.214	−0.031	−0.431	0.282	0.036
	*p*	0.859	0.998	0.913	0.777	0.362	0.739	0.969	0.949	0.965	0.984	0.157	0.782	0.482	0.92	0.141	0.35	0.907
Duration	*r*	0.169	−0.099	0.103	0.073	0.139	−0.212	0.182	0.031	0.176	−0.339	−0.362	−0.309	−0.265	−0.405	−0.536	−0.497	−0.508
	*p*	0.58	0.749	0.737	0.813	0.65	0.487	0.552	0.921	0.565	0.258	0.224	0.304	0.381	0.169	0.059	0.084	0.076
CAG	*r*	−0.046	−0.276	0.105	−0.31	−0.112	−0.025	−0.018	0.066	0.297	−0.139	−0.121	−0.047	0.174	−0.447	−0.14	−0.309	−0.083
	*p*	0.881	0.361	0.734	0.302	0.715	0.936	0.953	0.831	0.325	0.65	0.693	0.879	0.571	0.125	0.649	0.304	0.788
SARA	*r*	0.055	−0.433	−0.122	−0.353	0.201	−0.199	0.022	−0.231	0.156	−0.364	−0.167	−0.154	0.039	−0.512	−0.186	−0.316	0.226
	*p*	0.858	0.139	0.692	0.237	0.511	0.515	0.944	0.448	0.611	0.221	0.585	0.614	0.898	0.074	0.544	0.293	0.457
ICARS	*r*	0.096	**−0.714**	0.11	**−0.649**	0.398	−0.29	0.025	0.068	−0.008	−0.15	−0.248	−0.177	−0.32	−0.493	−0.376	−0.271	0.206
	*p*	0.754	**0.006**	0.721	**0.016**	0.178	0.336	0.935	0.826	0.978	0.624	0.414	0.563	0.286	0.087	0.205	0.371	0.499
MMSE	*r*	−0.128	0.208	−0.218	0.198	−0.098	0.057	−0.133	−0.205	−0.287	0.138	0.098	0.269	0.058	0.454	0.076	**0.593**	0.087
	*p*	0.676	0.494	0.475	0.516	0.749	0.854	0.666	0.501	0.342	0.653	0.749	0.373	0.852	0.119	0.805	**0.033**	0.778

Bold values indicate the statistically significant differences in the correlations (*p* < 0.05).

Additionally, the Stearoyl-CoA desaturase (SCD) indices (FFA 16:1/16:0, FFA 18:1/18:0) analysis showed increased SCD indices in the symptomatic SCA3 group as compared with the Pre-SCA3 group and control group (*P* < 0.001; Table 4).

### Perturbed Metabolic Pathways

**Figure 2:**
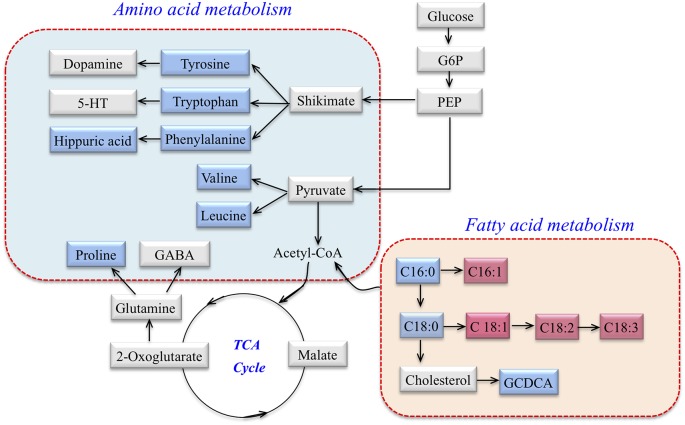
Metabolic network of differential metabolites involved in amino acid (AA) metabolism and fatty acid metabolism in SCA3 patients. Blue: downregulation in SCA3 patients. Pink: up-regulation in SCA3 patients. Gray: no change.

According to MetaboAnalyst 3.0, HMDB, KEGG, a map of the altered metabolic pathways for SCA3 patients was constructed (Figure 2). The serum metabolic profiling changed with the progression of the disease and was mainly associated with AA and fatty acid metabolism pathways.

### Potential Biomarkers

**Figure 3:**
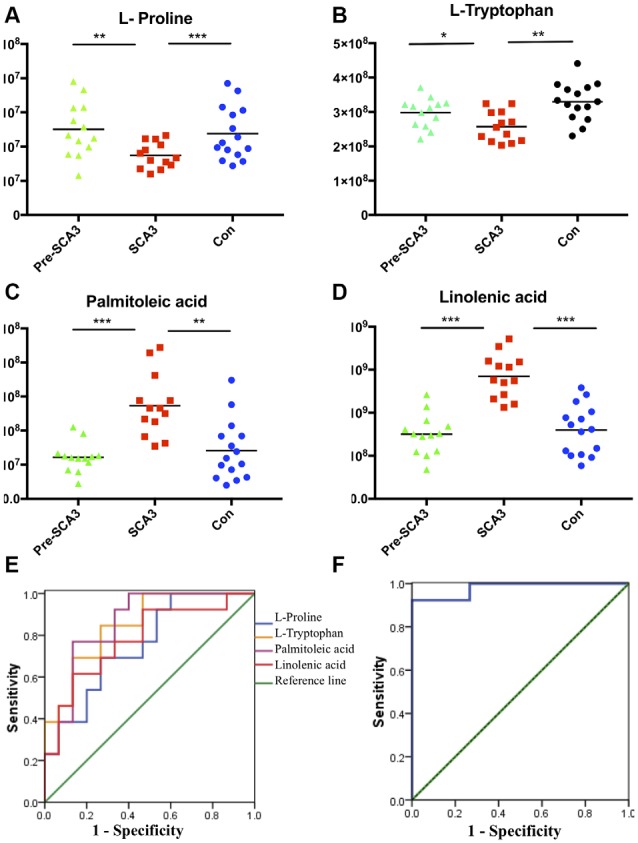
The scatter charts and ROC curves of candidate biomarkers. **(A–D)** The scatter charts of the L-Proline, L-Tryptophan, palmitoleic acid (FFA 16:1), linolenic acid (FFA 18:3) in the three groups. **(E)** ROC curves of the four candidate diagnostic biomarkers to distinguish symptomatic SCA3 patients and controls. **(F)** Combined diagnosis ROC curve of the combined four candidate biomarkers. *P < 0.05, **P < 0.01, ***P < 0.001.

The ROC curves were plotted based on the differential metabolites between symptomatic SCA3 group and control group. Metabolites with area under curve (AUC) >0.7 were selected as potential biomarkers. FFA 16:1 (palmitoleic acid), FFA 18:3 (linolenic acid), L-Proline and L-Tryptophan were identified as potential biomarkers (Figures 3A–E). A combined ROC analysis was performed with the selected biomarkers, followed by binary logistic regression analysis, with AUC reaching 0.979 (Figure 3F, Table 5).

**Table 4:** SCD indices analysis in the three groups.

SCD indices	Pre-SCA3	SCA3	Con	*P^a^*	SCA3 vs. Con		SCA3 vs. Pre	
					*P^b^*	FC^c^	*P^b^*	FC^c^
16:1/16:0	0.073 (0.022)	0.177 (0.063)	0.085 (0.055)	<0.001	<0.001	2.08	<0.001	2.42
18:1/18:0	1.448 (0.561)	4.604 (1.806)	1.673 (0.927)	<0.001	<0.001	2.75	<0.001	3.18

*^a^*ANOVA*. ^b^*t*-test. ^c^FC: fold change, calculated from the arithmetic mean values of each group*.

**Table 5:** The Receive Operating Characteristic (ROC) curve analysis for the candidate diagnostic biomarkers.

Metabolites	AUC	SE	95%CI	*P*	Sensitivity (%)	Specificity (%)
FFA 16:1	0.856	0.072	0.715–0.998	0.001	0.769	0.867
FFA 18:1	0.779	0.090	0.604–0.955	0.012	0.615	0.867
L-Proline	0.754	0.091	0.575–0.932	0.023	0.733	0.692
L-Tryptophan	0.851	0.071	0.712–0.990	0.002	0.733	0.846
Combined	0.979	0.023	0.934–1.000	<0.001	0.923	1.000

*AUC, area under curve; SE, standard error of mean; CI, confidence interval; Combined: FFA16:1 + FFA18:1 + L-Proline + L-Tryptophan*.

## Discussion

This study presents the first evaluation of the serum metabolomic profile of patients with SCA3. Our results show that the metabolomic profile of symptomatic SCA3 patients differs significantly from preclinical SCA3 patients and healthy controls, while the metabolomic profile of preclinical SCA3 patients shows no obvious difference compared to healthy controls. Importantly, the differential metabolites of symptomatic SCA3 patients revealed perturbations in AA metabolism and fatty acid metabolism.

The AA metabolic pathway was found to be significantly disrupted in the symptomatic SCA3 group. Branched-chain amino acids (BCAAs) including valine and leucine, and aromatic amino acids (ArAAs) including tryptophan and tyrosine, were all downregulated in the serum of SCA3 patients. Moreover, proline and the product of phenylalanine, hippuric acid, were also decreased. With respect to the perturbed biochemical pathways, these altered metabolites are not only related to energy metabolism, but also influence the metabolism of neurotransmitters. We inferred that 5-hydroxytryptamine (5-HT), dopamine, and γ-aminobutyric acid (GABA) may also be affected in SCA3 patients. Indeed, previous studies have suggested that dopamine and 5-HT pathways are associated with SCA3 (Schols et al., 1998, 2015; Takei et al., 2002, 2005; Teixeira-Castro et al., 2015; Martinez et al., 2017). Besides, BCAAs were associated with lowered 5-HT levels because BCAAs can compete with tryptophan (a precursor of 5-HT) for transportation across the blood-brain barrier (Choi et al., 2013).

Interestingly, a recent metabolomic study of plasma from a mouse model of SCA3 reported tryptophan as the most promising biomarker, which is consistent with our results (Toonen et al., 2018). Tryptophan has also been reported to be elevated in Huntington disease because of increased 3-hydroxyanthranilate oxygenase activity (Schwarcz et al., 1988). Thus, the detection of tryptophan and 3-hydroxyanthranilate oxygenase in SCA3 patients corroborates our results.

Fatty acid metabolism was another significantly perturbed pathway in symptomatic SCA3 patients. β-oxidation of free fatty acid (FFA) is a multi-step process in which fatty acids are broken down in various tissues to produce energy. In this study, the level of saturated fatty acid (SFA) decreased in the serum of symptomatic SCA3 patients, whereas that of monounsaturated fatty acid (MUFA) and polyunsaturated fatty acid (PUFA) increased. Specifically, palmitic acid (FFA 16:0) and stearic acid (FFA 18:0) were decreased, while palmitoleic acid (FFA 16:1), oleic acid (FFA 18:1), linoleic acid (FFA 18:2) and linolenic acid (FFA 18:3) were increased. SCD is the key enzyme that catalyzes the conversion of saturated fatty acids to unsaturated fatty acids, especially for FFA 16:0 and FFA 18:0. The results indicated two-fold elevated SCD indices in symptomatic SCA3 patients. Indeed, previous studies have shown that SCD indices are elevated in some diseases, such as Alzheimer’s disease and Amyotrophic Lateral Sclerosis (Astarita et al., 2011; Henriques et al., 2015). However, the role of SCD in SCA3 patients still needs to be elucidated in order to explain our results.

Glycochenodeoxycholate (GCDCA) is a conjugated bile acid, composed of glycine and chenodeoxycholic acid, which is associated with cholesterol metabolism (Li and Chiang, 2009). The carnitine system, including free carnitine and acylcarnitines, is essential for cellular energy metabolism as a carrier of long-chain fatty acids for β-oxidation or as a reservoir of acyl-CoA (Jones et al., 2010). Hippuric acid (N-Benzoylglycine) is synthesized from benzoic acid and glycine by the enzyme acyl-CoA:glycine N-acyltransferase (GLYAT; Irwin et al., 2016). The decrease of hippuric acid in SCA3 patients indicates the abnormal AA and fatty acid metabolism of SCA3 patients. However, it is unclear whether the key enzyme, GLYAT, functions abnormally.

According to the ROC curve analysis, a set of four candidate biomarkers, composed of FFA 16:1, FFA 18:3, L-Proline and L-Tryptophan, were selected to differentiate the symptomatic SCA3 patients from the healthy controls with an AUC value of 0.979. These potential metabolite markers provide a novel and promising diagnostic approach for detection of SCA3.

However, there are limitations in this study. The chemical concentration of the metabolites in blood may weakly represent the concentration in the brain, and obtaining CSF or brain tissue may be invasive and unethical. The number of samples is another limitation as this is an exploratory study designed to evaluate the metabolic profiles of age-, sex-, BMI-matched preclinical and symptomatic SCA3 patients. Another limitation is that it is a cross-sectional study, which may only represent short-term metabolic perturbations. However, the data serves as a foundation for future longitudinal studies.

SCA3 is a slow progressing disorder with a long preclinical period, and the development of molecular biomarkers is urgently needed. Recently, promising candidate molecular biomarkers of SCA3 have emerged, including genetic modifiers, transcriptional biomarkers, and mitochondrial DNA damage (Franca et al., 2012; Raposo et al., 2015a,b, 2017, 2019; Ramos et al., 2019). In our study, although not being able to differentiate between preclinical carriers and symptomatic patients, these biomarkers should be crucial to improve sensitivity, when used in complement to clinical and imaging markers. Furthermore, when ameliorating drugs will be available, these biomarkers being able to detect pathogenic alterations will be useful to optimize therapeutics efficiency.

In conclusion, the serum metabolic profiling is altered with the progress of the disease in SCA3 patients, the perturbations being mainly associated with AA metabolism and fatty acid metabolism pathways. A panel of four biomarkers that confidently detect the disease is proposed as promising biomarkers. However, further longitudinal studies on larger cohorts, especially those designed to investigate changes in neurotransmitters and metabolomic profiles in CSF or cerebellum of SCA3 patients, are required to validate our findings.

## Data Availability

All datasets generated for this study are included in the manuscript and/or the Supplementary Files.

## Ethics Statement

This study was carried out in accordance with the recommendations of the Ethics Committee of the First Affiliated Hospital of Zhengzhou University with written informed consent from all subjects. All subjects gave written informed consent in accordance with the Declaration of Helsinki. The protocol was approved by the Ethics Committee of the First Affiliated Hospital of Zhengzhou University.

## Author Contributions

YX and GX designed the project and reviewed the article. ZY, CS and LZ performed the metabolomic analysis, data analysis, and drafted the manuscript. YLi revised the manuscript. JY and YLiu assessed the clinical characteristics of the subjects. CM and HL collected the serum.

## Supplementary Material

The Supplementary Material for this article can be found online at: https://www.frontiersin.org/articles/10.3389/fnmol.2019.00159/full#supplementary-material

FIGURE S1 The relative standard deviation (RSD) distribution of ions detected in each metabolomics analytical platform. **(A)** UHPLC-MS in negative mode. **(B)** UHPLC-MS in positive mode. **(C)** GC-MS. Data are normalized to the sum of the peaks detected in each platform, respectively.

FIGURE S2 Typical total ion chromatograms (TIC) from the quality control (QC) Sample. **(A)** UHPLC-MS in negative mode. **(B)** UHPLC-MS in positive mode. **(C)** GC-MS.

TABLE S1 Metabolites identified from GC-MS and UHPLC-MS (ESI+, ESI−) platforms. RT: Retention time; MZ: Mass/charge ratio; ESI+: Electrospray ionization positive mode; ESI−: Electrospray ionization negative mode.

## Conflict of Interest Statement

The authors declare that the research was conducted in the absence of any commercial or financial relationships that could be construed as a potential conflict of interest.

## Footnotes

^1^https://www.metaboanalyst.ca

^2^http://www.hmdb.ca

^3^https://www.kegg.jp
