# Supplementary figures and images for: Metabolic Profiling Reveals Biochemical Pathways and Potential Biomarkers of Spinocerebellar Ataxia 3
Source: Front Mol Neurosci. 2019 Jun 27;12:159. doi: 10.3389/fnmol.2019.00159 (PMC6611058; doi:10.3389/fnmol.2019.00159)

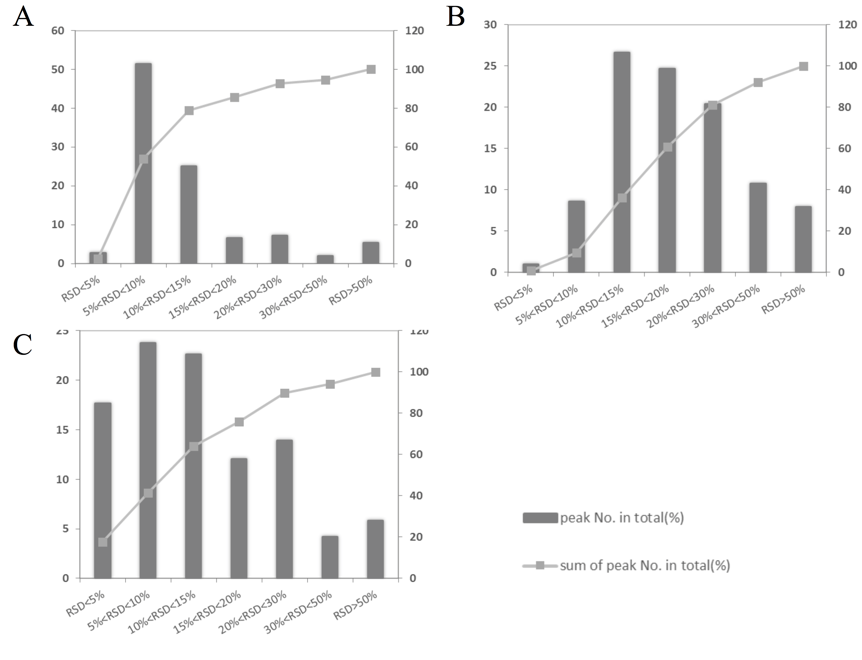

Supplement: FIGURE S1 — The relative standard deviation (RSD) distribution of ions detected in each metabolomics analytical platform. (A) UHPLC-MS in negative mode. (B) UHPLC-MS in positive mode. (C) GC-MS. Data are normalized to the sum of the peaks detected in each platform, respectively. [file Image_1.TIFF]

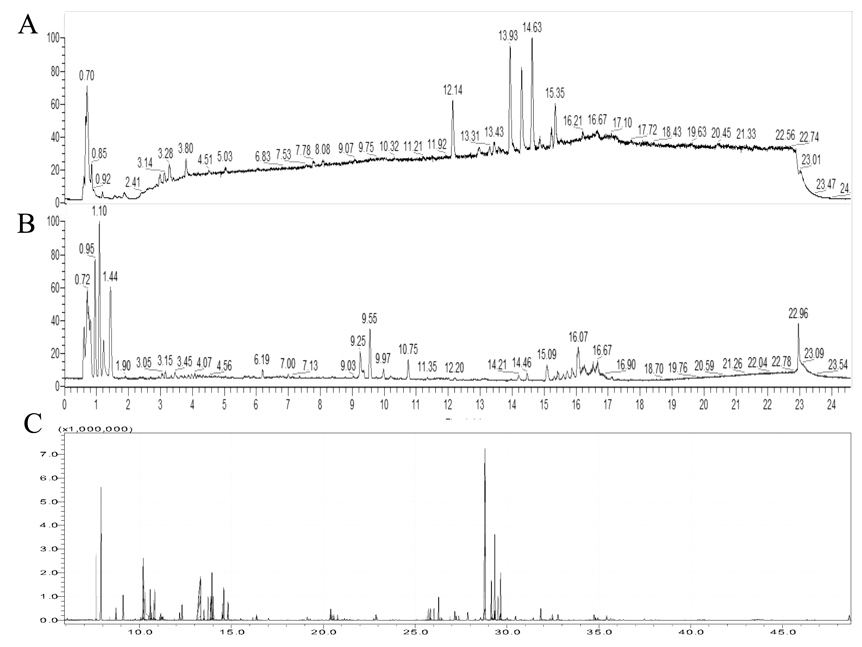

Supplement: FIGURE S2 — Typical total ion chromatograms (TIC) from the quality control (QC) Sample. (A) UHPLC-MS in negative mode. (B) UHPLC-MS in positive mode. (C) GC-MS. [file Image_2.TIFF]
